# Synthesis and structure of the mercury chloride complex of 2,2′-(2-bromo-5-*tert*-butyl-1,3-phenyl­ene)bis­(1-methyl-1*H*-benzimidazole)

**DOI:** 10.1107/S2056989017001888

**Published:** 2017-02-10

**Authors:** Varsha Rani, Harkesh B. Singh, Ray J. Butcher

**Affiliations:** a Department of Chemistry, Indian Institute of Technology Bombay, Powai, Mumbai 400 076, India; b Department of Chemistry, Howard University, 525 College Street NW, Washington, DC 20059, USA

**Keywords:** crystal structure, mercury coordination polymer, (benzimidazol-2-yl)benzene ligands

## Abstract

In the title mercury complex, *catena*-poly[[di­chlorido­mercury(II)]-μ-2,2′-(2-bromo-5-*tert*-butyl-1,3-phenyl­ene)bis­(1-methyl-1*H*-benzimidazole)-κ^2^ *N* ^3^:*N* ^3′^], [HgCl_2_(C_26_H_25_BrN_4_)]_*n*_, the Hg^II^ atom is coordinated by two Cl atoms and by two N atoms from two 2,2′-(2-bromo-5-*tert*-butyl-1,3-phenyl­ene)bis­(1-methyl-1*H*-benzimidazole) ligands. The metal cation adopts a distorted tetrahedral coordination geometry with with bond angles around mercury of 100.59 (15)° [N—Hg—N] and 126.35 (7)° [Cl—Hg—Cl]. This arrangement gives rise to a zigzag helical 1-D polymer propagating along the *b-*axis direction.

## Abstract 2

In the title mercury complex, the Hg^II^ atom is coordinated by two Cl atoms and by two N atoms from two 2,2′-(2-bromo-5-*tert*-butyl-1,3-phenyl­ene)bis­(1-methyl-1*H*-benzimidazole) ligands, which gives rise to a zigzag helical 1-D polymer propagating along the *b*-axis direction.

## Chemical context   

In the last one decade, 1,3-bis­(benzimidazol-2-yl)benzene-based ligands have been studied extensively due to the presence of active sites for binding of metal atoms (Yang *et al.*, 2012 ▸; Tam *et al.*, 2011 ▸; Dorazco-Gonzalez, 2014 ▸). Very recently, dinuclear zinc complexes containing (benzimidazol-2-yl)benzene-based ligands have shown remarkable anti­cancer activities (Xie *et al.*, 2014 ▸). Helical and non-helical complexes with copper(I) have been reported by Ruettimann *et al.* (1992 ▸). Palladium complexes with bromo-functionalized benzimid­azole derivatives have been utilized for Heck reactions (Reddy & Krishna, 2005 ▸).

A survey of the structural investigations of mercury halide complexes with benzimidazole derivatives have shown that they come in two main types, *viz*. polymeric, bridging either through the halide (Zhang *et al.*, 2015 ▸; Li *et al.*, 2007 ▸; Shen *et al.*, 2005 ▸) or through alternative N atoms from the benzimidazole moieties (Xiao *et al.*, 2009 ▸, 2011 ▸; Huang *et al.*, 2006 ▸; Li *et al.*, 2007 ▸, 2012*a* ▸,*b* ▸; Dey *et al.*, 2013 ▸; Du *et al.*, 2011 ▸; Chen *et al.*, 2013 ▸; Su *et al.*, 2003 ▸; Xu *et al.*, 2011 ▸), or discrete mol­ecules, *i.e.* non-polymeric (Xiao *et al.*, 2011 ▸; Wu *et al.*, 2009 ▸; Zhao *et al.*, 2012 ▸; Lou *et al.*, 2012 ▸; Zhu *et al.*, 2009 ▸; Carballo *et al.*, 1993 ▸; Yan *et al.*, 2012 ▸; Hu *et al.*, 2012 ▸, 2015 ▸; Ding *et al.*, 2012 ▸; Matthews *et al.*, 1998 ▸; Manjunatha *et al.*, 2011 ▸; Wang *et al.*, 2007 ▸, 2009 ▸, 2012 ▸, 2015 ▸; Chen *et al.*, 2014 ▸; Su *et al.*, 2003 ▸; Quiroz-Castro *et al.*, 2000 ▸; Yang & Luo, 2012 ▸; He *et al.*, 2012 ▸; Bouchouit *et al.*, 2015 ▸).

**Figure 1:**
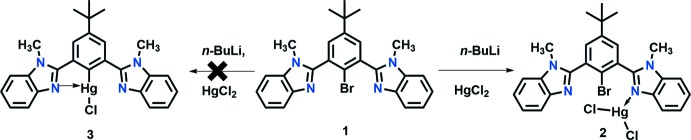
Diagram showing the the starting compound, **1**, the title compound, **2**, and the expected product, **3**.

In the present case, during the attempted synthesis of the C-2 mercurated derivative **3** from 2,2′-(2-bromo-5-*tert*-butyl-1,3-phenyl­ene)bis­(1-methyl-1*H*-benzimidazole), **1**, using *n*-BuLi and mercuric chloride, the mercury complex **2** was isolated unexpectedly (Fig. 1 ▸).

## Structural commentary   

The structure of **2** with empirical formula, C_26_H_25_BrCl_2_HgN_4_, is reported in this paper. As a result of the presence of the Br and *t*-butyl substituents on the central ring, coordination of the Hg^II^ atom to this ring is prevented and thus a monomeric complex is formed, as has previously been observed for an HgCl_2_ complex with a similar ligand but with a central pyridine ring rather than a phenyl ring (Liu *et al.*, 2007 ▸).

**Figure 2:**
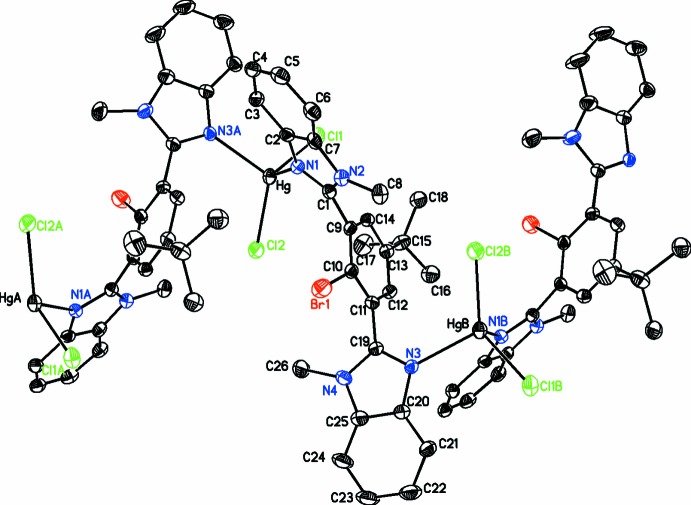
Diagram showing the three units which assemble to form a coordination polymer and illustrating its zigzag helical nature (with H atoms omitted for clarity). Displacement parameters are drawn at the 30% probability level. [Symmetry codes: (*A*) 1 − *x*, 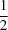 + *y*, *z* − 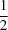; (*B*) 1 − *x*, *y* − 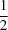, *z* − 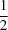.]

Another related structure has recently been reported of a dinuclear structure of HgCl_2_ with a similar ligand to **1** where there is a methyl substituent on the C1 atom of the imidazole ring (Hu *et al.*, 2015 ▸). In the case of **2**, however, a zigzag polymeric structure forms in the *b*-axis direction, in which the HgCl_2_ moiety is linked by atoms N1 from one ligand and N3 from an adjoining ligand. The coordination environment around the mercury atom is distorted tetra­hedral with bond angles ranging from 100.6 (2) to 126.35 (7)° (Fig. 2 ▸). The two Hg—N bond lengths are equivalent at 2.333 (4) and 2.338 (4) Å. However, the metal–halogen bonds are not similar [Hg—Cl1 = 2.4424 (13) and Hg—Cl2 = 2.4020 (15) Å]. The ligand adopts a conformation whereby the two benzimidazole moieties are not coplanar with each other or the central phenyl ring. The dihedral angles between the benzimidazole moieties N1/N2/C1–C7 and N3/N4/C19–C24 are 60.9 (2)° while they make dihedral angles of 55.6 (2) and 84.2 (2)°, respectively, with the central ring.
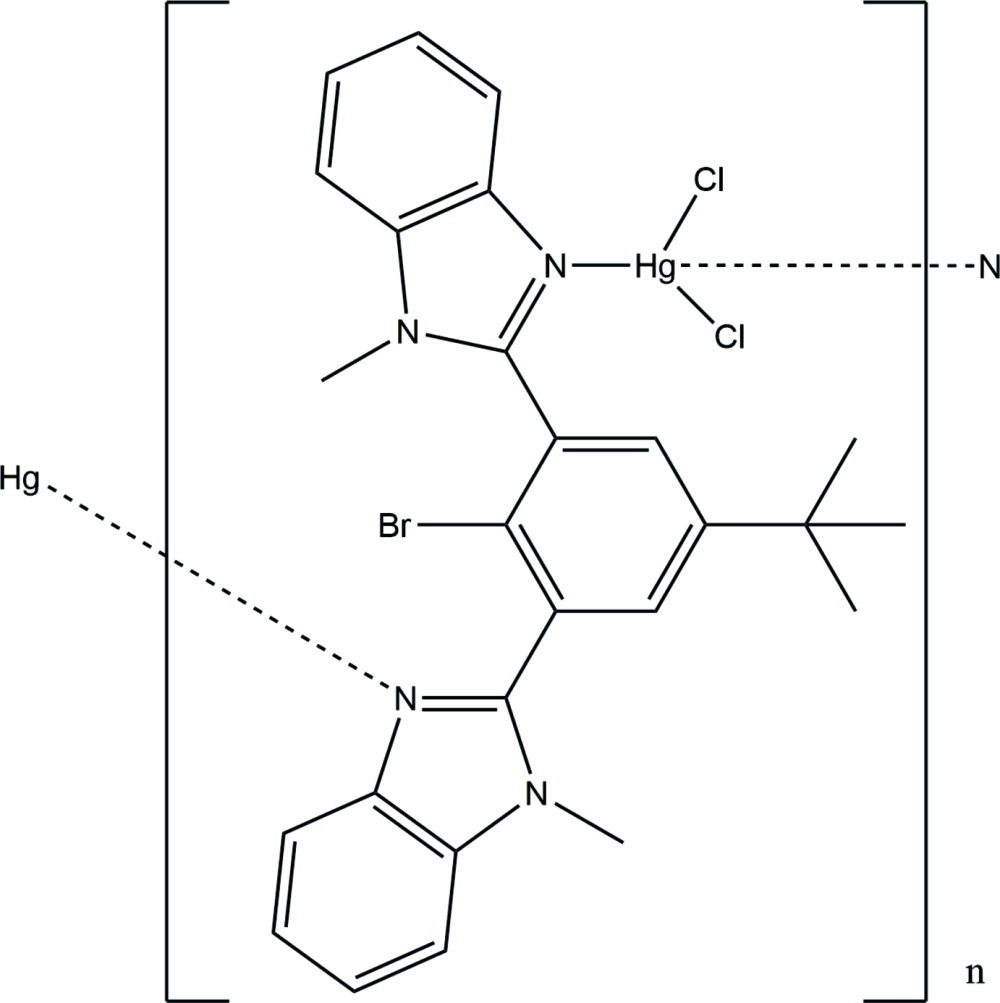


## Supra­molecular features   

**Figure 3:**
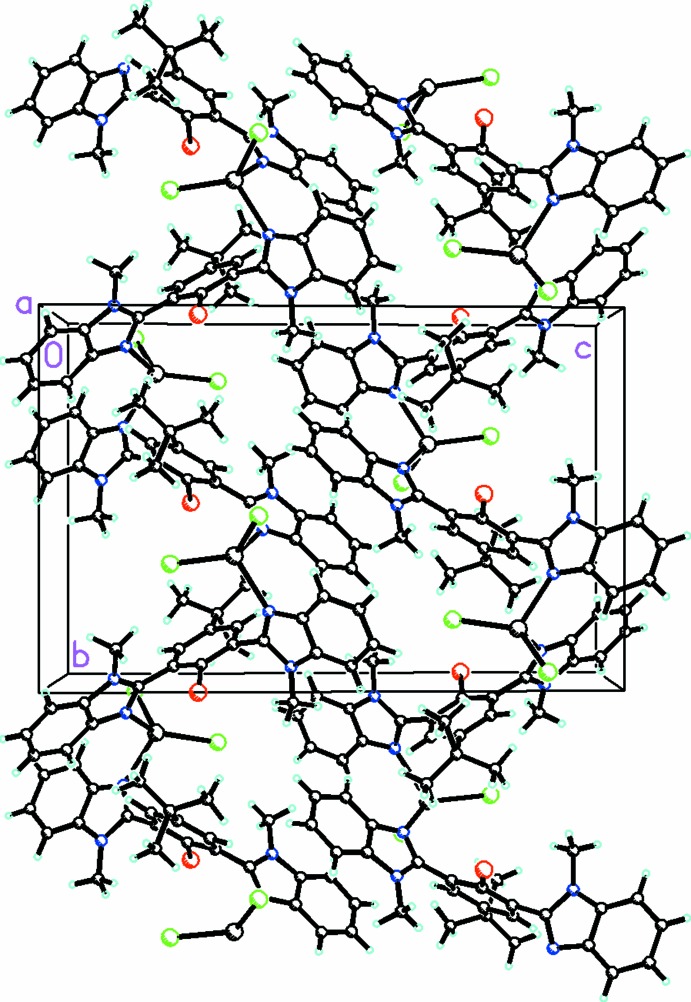
Packing diagram showing two units of the polymer, which repeat in the *b*-axis direction, viewed along the *a* axis.

The combination of HgCl_2_ with 2,2′-(2-bromo-5-*tert*-butyl-1,3-phenyl­ene)bis­(1-methyl-1*H*-benzimidazole) results in a zigzag helical 1-D coordination polymer that propagates along the *b-*axis direction. This is mediated by the HgCl_2_ moiety, which is linked by atoms N1 from one ligand and N3 from an adjoining ligand (Fig. 2 ▸). Although helices are inherently chiral in nature, the overall structure is not chiral as the individual helices are related by a center of inversion. The inter­nal structure of this polymer is stabilized by both C—H⋯Cl and C—H⋯N inter­actions (Table 1 ▸). In addition, there are both C—H⋯π (Table 1 ▸) and π–π inter­actions [*Cg*6⋯*Cg*6(1 − *x*, −*y*, −*z*) = 3.531 (2) Å, where *Cg*6 is the centroid of the benzimidazole ring system N3/N4/C19–C24 and C25]. There are no halogen bonds or C—H⋯Br inter­actions present. Apart from van der Waals inter­actions, there are no significant inter­actions between the zigzag chains of the coordination polymer (Fig. 3 ▸).

## Database survey   

A search of the Cambridge Structural Database (Version 5.37 with updates May 2016; Groom *et al.*, 2016 ▸) reveals that there is no report in the literature for a mercury complex with 2,2′-(2-bromo-5-*tert*-butyl-1,3-phenyl­ene)bis­(1-methyl-1*H*-benzimidazole) that has been structurally characterized. A cadmium complex, bis­[1,3-bis­(benzimidazol-2-yl)benzene]­dichlorido­cadmium(II), in which the Cd is coordinated by two Cl atoms and two N atoms in a distorted tetra­hedral configuration has been reported (Jiang *et al.*, 2010 ▸). In the title complex **2**, cadmium is replaced by an Hg^II^ atom along with a slight modification of the ligand.

## Synthesis and crystallization   

To a solution of **1** (0.2 g, 0.42 mmol) in THF (15 ml) was added dropwise a solution of *n*-BuLi (0.3 ml, 0.47 mmol) at 195 K. The synthesis of compound **1** will be published elsewhere. The reaction mixture turned blue after immediate addition of *n*-BuLi. The reaction mixture was stirred for 30 min at 195 K followed by the addition of HgCl_2_ (0.126 g, 0.466 mmol). The reaction mixture was warmed to room temperature and stirred for 16 h. The reaction mixture was then filtered through Whatman filter paper and the solvent was evaporated on a rotary evaporator. Colourless plate-shaped crystals were obtained by the slow evaporation of an ethyl acetate solution of the compound at room temperature.

Yield 44% (0.138 g), ^1^H NMR (400 MHz, CDCl_3_): δ 7.88–7.86 (*m*, 3H), 7.45–7.34 (*m*, 7H), 3.98 (*s*, 6H), 1.46 (*s*, 9H). ^13^C NMR (100 MHz, DMSO): 152.3, 151.2, 141.6, 135.2, 131.8, 131.4, 123.3, 122.7, 121.6, 119.1, 111.0, 34.9, 31.1, 30.8. Analysis calculated for C_26_H_25_N_4_Cl_2_BrHg: C, 41.92; H, 3.38; N, 7.52. Found C, 42.68; H, 4.14; N, 6.29.

## Refinement   

Crystal data, data collection and structure refinement details are summarized in Table 2 ▸. H atoms were positioned geomet­ric­ally and refined as riding: C—H = 0.95–0.98 Å with *U*_iso_(H) = 1.2*U*_eq_(C) or 1.5*U*_eq_(C) for methyl H atoms.

## Supplementary Material

Crystal structure: contains datablock(s) I. DOI: 10.1107/S2056989017001888/zl2694sup1.cif

Structure factors: contains datablock(s) I. DOI: 10.1107/S2056989017001888/zl2694Isup2.hkl

CCDC reference: 1530778

Additional supporting information:  crystallographic information; 3D view; checkCIF report

## Figures and Tables

**Table 1:** Hydrogen-bond geometry (Å, °) *Cg*1 is the centroid of the imidazole ring N1/N2/C1/C2/C7.

*D*—H⋯*A*	*D*—H	H⋯*A*	*D*⋯*A*	*D*—H⋯*A*
C3—H3*A*⋯N3^i^	0.95	2.65	3.459 (7)	144
C8—H8*A*⋯Cl2^ii^	0.98	2.71	3.643 (6)	160
C8—H8*B*⋯Cl1^iii^	0.98	2.82	3.719 (6)	152
C21—H21*B*⋯Cl1^ii^	0.95	2.77	3.616 (3)	149
C16—H16*B*⋯*Cg*1^ii^	0.98	2.91	3.671 (8)	135

Symmetry codes: (i) 
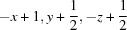
; (ii) 
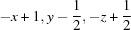
; (iii) 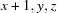.

**Table 2:** Experimental details

Crystal data	
Chemical formula	[HgCl_2_(C_26_H_25_BrN_4_)]
*M*_r_	744.90
Crystal system, space group	Monoclinic, *P*2_1_/*c*
Temperature (K)	123
*a*, *b*, *c* (Å)	9.50481 (18), 13.3872 (2), 20.3322 (4)
β (°)	93.0955 (19)
*V* (Å^3^)	2583.36 (9)
*Z*	4
Radiation type	Cu *K*α
μ (mm^−1^)	14.57
Crystal size (mm)	0.37 × 0.09 × 0.03

Data collection	
Diffractometer	Agilent Xcalibur, Ruby, Gemini
Absorption correction	Analytical [*CrysAlis PRO* (Agilent, 2012 ▸) based on expressions derived by Clark & Reid (1995 ▸)]
*T*_min_, *T*_max_	0.331, 1.000
No. of measured, independent and observed [*I* > 2σ(*I*)] reflections	9778, 5217, 4596
*R*_int_	0.034
(sin θ/λ)_max_ (Å^−1^)	0.628

Refinement	
*R*[*F* ^2^ > 2σ(*F* ^2^)], *wR*(*F* ^2^), *S*	0.038, 0.104, 1.07
No. of reflections	5217
No. of parameters	300
H-atom treatment	H-atom parameters constrained
Δρ_max_, Δρ_min_ (e Å^−3^)	1.34, −1.88

Computer programs: *CrysAlis PRO* (Agilent, 2012 ▸), *SHELXS2013* (Sheldrick, 2008 ▸), *SHELXL2016* (Sheldrick, 2015 ▸) and *SHELXTL* (Sheldrick, 2008 ▸).

## supplementary crystallographic information

### Crystal data

**Table:**

[HgCl_2_(C_26_H_25_BrN_4_)]	*F*(000) = 1432
*M**_r_* = 744.90	*D*_x_ = 1.915 Mg m^−^^3^
Monoclinic, *P*2_1_/*c*	Cu *K*α radiation, λ = 1.54184 Å
*a* = 9.50481 (18) Å	Cell parameters from 4457 reflections
*b* = 13.3872 (2) Å	θ = 3.9–74.8°
*c* = 20.3322 (4) Å	µ = 14.57 mm^−^^1^
β = 93.0955 (19)°	*T* = 123 K
*V* = 2583.36 (9) Å^3^	Plate, colorless
*Z* = 4	0.37 × 0.09 × 0.03 mm

### Data collection

**Table:**

Agilent Xcalibur, Ruby, Gemini diffractometer	5217 independent reflections
Radiation source: Enhance (Cu) X-ray Source	4596 reflections with *I* > 2σ(*I*)
Detector resolution: 10.5081 pixels mm^-1^	*R*_int_ = 0.034
ω scans	θ_max_ = 75.6°, θ_min_ = 4.0°
Absorption correction: analytical [CrysAlis PRO (Agilent, 2012) based on expressions derived by Clark & Reid (1995)]	*h* = −11→11
*T*_min_ = 0.331, *T*_max_ = 1.000	*k* = −10→16
9778 measured reflections	*l* = −25→20

### Refinement

**Table:**

Refinement on *F*^2^	0 restraints
Least-squares matrix: full	Hydrogen site location: inferred from neighbouring sites
*R*[*F*^2^ > 2σ(*F*^2^)] = 0.038	H-atom parameters constrained
*wR*(*F*^2^) = 0.104	*w* = 1/[σ^2^(*F*_o_^2^) + (0.0583*P*)^2^] where *P* = (*F*_o_^2^ + 2*F*_c_^2^)/3
*S* = 1.07	(Δ/σ)_max_ = 0.001
5217 reflections	Δρ_max_ = 1.34 e Å^−^^3^
300 parameters	Δρ_min_ = −1.88 e Å^−^^3^

### Special details

**Table:**

Geometry. All esds (except the esd in the dihedral angle between two l.s. planes) are estimated using the full covariance matrix. The cell esds are taken into account individually in the estimation of esds in distances, angles and torsion angles; correlations between esds in cell parameters are only used when they are defined by crystal symmetry. An approximate (isotropic) treatment of cell esds is used for estimating esds involving l.s. planes.

### Fractional atomic coordinates and isotropic or equivalent isotropic displacement parameters (Å^2^)

**Table:**

	*x*	*y*	*z*	*U*_iso_*/*U*_eq_	
Hg	0.26669 (2)	0.15196 (2)	0.17398 (2)	0.03689 (9)	
Br1	0.78523 (7)	0.01223 (6)	0.26403 (4)	0.05807 (19)	
Cl1	0.07681 (12)	0.04507 (10)	0.12894 (8)	0.0455 (3)	
Cl2	0.3350 (3)	0.17449 (13)	0.28849 (7)	0.0680 (5)	
N1	0.4632 (4)	0.0840 (3)	0.12648 (19)	0.0297 (8)	
N2	0.6469 (4)	−0.0168 (3)	0.1125 (2)	0.0332 (8)	
C1	0.5402 (5)	0.0084 (4)	0.1512 (2)	0.0290 (9)	
C2	0.5243 (5)	0.1104 (4)	0.0687 (2)	0.0325 (10)	
C3	0.4867 (6)	0.1831 (4)	0.0214 (2)	0.0380 (11)	
H3A	0.407599	0.225411	0.026176	0.046*	
C4	0.5690 (7)	0.1912 (5)	−0.0326 (3)	0.0443 (13)	
H4A	0.546152	0.239743	−0.065535	0.053*	
C5	0.6869 (7)	0.1279 (5)	−0.0392 (3)	0.0462 (13)	
H5A	0.742376	0.136244	−0.076319	0.055*	
C6	0.7235 (6)	0.0557 (4)	0.0054 (3)	0.0415 (12)	
H6A	0.801480	0.012676	−0.000153	0.050*	
C7	0.6402 (5)	0.0478 (4)	0.0599 (2)	0.0336 (10)	
C8	0.7432 (5)	−0.1007 (4)	0.1193 (3)	0.0412 (11)	
H8A	0.706934	−0.149329	0.150248	0.062*	
H8B	0.835960	−0.077071	0.136067	0.062*	
H8C	0.751825	−0.132519	0.076325	0.062*	
C9	0.5103 (5)	−0.0449 (4)	0.2128 (2)	0.0295 (9)	
C10	0.6114 (5)	−0.0530 (4)	0.2651 (2)	0.0328 (9)	
C11	0.5801 (5)	−0.1064 (4)	0.3208 (2)	0.0347 (10)	
C12	0.4486 (6)	−0.1492 (4)	0.3257 (2)	0.0340 (10)	
H12A	0.429536	−0.186816	0.363769	0.041*	
C13	0.3436 (5)	−0.1382 (4)	0.2757 (2)	0.0326 (10)	
C14	0.3774 (5)	−0.0858 (4)	0.2192 (2)	0.0301 (9)	
H14A	0.307701	−0.078004	0.184253	0.036*	
C15	0.1946 (6)	−0.1774 (5)	0.2844 (3)	0.0446 (13)	
C16	0.1931 (7)	−0.2560 (6)	0.3375 (3)	0.0569 (16)	
H16A	0.250243	−0.313200	0.325066	0.085*	
H16B	0.096006	−0.277900	0.342884	0.085*	
H16C	0.232117	−0.227992	0.379161	0.085*	
C17	0.1086 (9)	−0.0864 (7)	0.3074 (4)	0.069 (2)	
H17A	0.014973	−0.108654	0.319073	0.103*	
H17B	0.098912	−0.037282	0.271679	0.103*	
H17C	0.157701	−0.055702	0.345930	0.103*	
C18	0.1272 (7)	−0.2131 (5)	0.2188 (3)	0.0504 (14)	
H18A	0.189918	−0.261007	0.198742	0.076*	
H18B	0.111504	−0.155783	0.189316	0.076*	
H18C	0.036859	−0.245312	0.226242	0.076*	
N3	0.7624 (4)	−0.2007 (3)	0.3870 (2)	0.0334 (8)	
N4	0.7052 (7)	−0.0512 (4)	0.4249 (3)	0.0574 (15)	
C19	0.6851 (6)	−0.1200 (4)	0.3764 (3)	0.0389 (11)	
C20	0.8389 (4)	−0.1846 (3)	0.44605 (14)	0.0393 (11)	
C21	0.9323 (4)	−0.2448 (2)	0.48302 (18)	0.0436 (12)	
H21B	0.958442	−0.308251	0.466606	0.052*	
C22	0.9875 (5)	−0.2123 (3)	0.54401 (18)	0.0604 (18)	
H22B	1.051354	−0.253523	0.569280	0.073*	
C23	0.9493 (6)	−0.1196 (4)	0.56803 (19)	0.085 (3)	
H23B	0.987023	−0.097343	0.609714	0.102*	
C24	0.8559 (6)	−0.0593 (3)	0.5311 (2)	0.092 (4)	
H24B	0.829779	0.004111	0.547474	0.111*	
C25	0.8007 (5)	−0.0918 (3)	0.4701 (2)	0.0562 (17)	
C26	0.6419 (11)	0.0482 (6)	0.4279 (4)	0.080 (3)	
H26D	0.560680	0.052189	0.396154	0.121*	
H26E	0.711628	0.098707	0.417059	0.121*	
H26F	0.610913	0.060333	0.472366	0.121*	

### Atomic displacement parameters (Å^2^)

**Table:**

	*U*^11^	*U*^22^	*U*^33^	*U*^12^	*U*^13^	*U*^23^
Hg	0.03625 (13)	0.03134 (14)	0.04333 (13)	0.00299 (7)	0.00463 (8)	0.00013 (8)
Br1	0.0463 (3)	0.0589 (4)	0.0671 (4)	−0.0147 (3)	−0.0146 (3)	0.0142 (3)
Cl1	0.0290 (5)	0.0381 (7)	0.0696 (8)	−0.0006 (5)	0.0036 (5)	0.0048 (6)
Cl2	0.1242 (16)	0.0414 (8)	0.0383 (6)	0.0101 (9)	0.0035 (8)	−0.0041 (6)
N1	0.0269 (18)	0.030 (2)	0.0319 (17)	−0.0004 (15)	0.0016 (14)	0.0021 (15)
N2	0.0281 (18)	0.034 (2)	0.0383 (19)	−0.0019 (16)	0.0042 (15)	−0.0003 (17)
C1	0.0243 (19)	0.030 (2)	0.033 (2)	−0.0040 (17)	0.0014 (16)	−0.0013 (18)
C2	0.034 (2)	0.034 (3)	0.0289 (19)	−0.0077 (19)	−0.0034 (17)	−0.0009 (19)
C3	0.044 (3)	0.034 (3)	0.035 (2)	−0.010 (2)	−0.002 (2)	0.005 (2)
C4	0.061 (3)	0.039 (3)	0.033 (2)	−0.018 (3)	−0.001 (2)	0.003 (2)
C5	0.051 (3)	0.053 (3)	0.035 (2)	−0.023 (3)	0.009 (2)	−0.006 (2)
C6	0.041 (3)	0.042 (3)	0.042 (2)	−0.011 (2)	0.010 (2)	−0.008 (2)
C7	0.032 (2)	0.032 (2)	0.037 (2)	−0.0111 (19)	0.0068 (18)	−0.0054 (19)
C8	0.030 (2)	0.037 (3)	0.057 (3)	0.002 (2)	0.008 (2)	−0.003 (2)
C9	0.030 (2)	0.026 (2)	0.032 (2)	0.0013 (17)	−0.0007 (17)	0.0030 (17)
C10	0.029 (2)	0.026 (2)	0.042 (2)	−0.0050 (18)	−0.0071 (18)	0.0024 (19)
C11	0.039 (2)	0.032 (3)	0.032 (2)	0.001 (2)	−0.0086 (18)	−0.0024 (19)
C12	0.039 (3)	0.032 (3)	0.030 (2)	0.0009 (19)	0.0011 (19)	0.0045 (18)
C13	0.031 (2)	0.036 (3)	0.032 (2)	0.0023 (19)	0.0047 (18)	−0.0002 (18)
C14	0.027 (2)	0.033 (2)	0.0302 (19)	0.0032 (18)	0.0007 (16)	−0.0002 (17)
C15	0.032 (3)	0.060 (4)	0.042 (3)	−0.003 (2)	0.006 (2)	0.015 (3)
C16	0.057 (4)	0.061 (4)	0.053 (3)	−0.016 (3)	0.005 (3)	0.009 (3)
C17	0.060 (4)	0.073 (5)	0.075 (5)	0.013 (4)	0.027 (3)	0.007 (4)
C18	0.047 (3)	0.050 (4)	0.054 (3)	−0.020 (3)	−0.004 (2)	0.011 (3)
N3	0.0333 (19)	0.027 (2)	0.0393 (19)	−0.0024 (16)	−0.0082 (16)	0.0031 (16)
N4	0.074 (4)	0.044 (3)	0.051 (3)	0.019 (3)	−0.027 (3)	−0.016 (2)
C19	0.045 (3)	0.034 (3)	0.037 (2)	−0.001 (2)	−0.011 (2)	0.003 (2)
C20	0.043 (3)	0.039 (3)	0.035 (2)	−0.001 (2)	−0.004 (2)	0.002 (2)
C21	0.042 (3)	0.042 (3)	0.045 (3)	0.004 (2)	−0.006 (2)	0.007 (2)
C22	0.055 (4)	0.077 (5)	0.048 (3)	0.012 (3)	−0.018 (3)	0.002 (3)
C23	0.106 (7)	0.090 (6)	0.055 (4)	0.031 (5)	−0.043 (5)	−0.027 (4)
C24	0.124 (8)	0.075 (6)	0.070 (5)	0.039 (5)	−0.057 (5)	−0.037 (4)
C25	0.070 (4)	0.049 (4)	0.048 (3)	0.013 (3)	−0.021 (3)	−0.005 (3)
C26	0.110 (7)	0.047 (4)	0.079 (5)	0.035 (4)	−0.042 (5)	−0.021 (4)

### Geometric parameters (Å, º)

**Table:**

Hg—N1	2.333 (4)	C13—C15	1.530 (7)
Hg—N3^i^	2.338 (4)	C14—H14A	0.9500
Hg—Cl2	2.4020 (15)	C15—C16	1.508 (8)
Hg—Cl1	2.4424 (13)	C15—C18	1.525 (8)
Br1—C10	1.870 (5)	C15—C17	1.553 (10)
N1—C1	1.331 (6)	C16—H16A	0.9800
N1—C2	1.383 (6)	C16—H16B	0.9800
N2—C1	1.359 (6)	C16—H16C	0.9800
N2—C7	1.374 (7)	C17—H17A	0.9800
N2—C8	1.452 (7)	C17—H17B	0.9800
C1—C9	1.483 (6)	C17—H17C	0.9800
C2—C3	1.401 (7)	C18—H18A	0.9800
C2—C7	1.404 (7)	C18—H18B	0.9800
C3—C4	1.387 (8)	C18—H18C	0.9800
C3—H3A	0.9500	N3—C19	1.318 (7)
C4—C5	1.417 (10)	N3—C20	1.387 (4)
C4—H4A	0.9500	N4—C19	1.355 (7)
C5—C6	1.358 (9)	N4—C25	1.369 (6)
C5—H5A	0.9500	N4—C26	1.463 (9)
C6—C7	1.401 (7)	C20—C21	1.3900
C6—H6A	0.9500	C20—C25	1.3900
C8—H8A	0.9800	C21—C22	1.3900
C8—H8B	0.9800	C21—H21B	0.9500
C8—H8C	0.9800	C22—C23	1.3900
C9—C14	1.389 (7)	C22—H22B	0.9500
C9—C10	1.398 (6)	C23—C24	1.3900
C10—C11	1.387 (7)	C23—H23B	0.9500
C11—C12	1.383 (8)	C24—C25	1.3900
C11—C19	1.478 (6)	C24—H24B	0.9500
C12—C13	1.392 (7)	C26—H26D	0.9800
C12—H12A	0.9500	C26—H26E	0.9800
C13—C14	1.399 (7)	C26—H26F	0.9800
			
N1—Hg—N3^i^	100.59 (15)	C16—C15—C18	112.8 (6)
N1—Hg—Cl2	105.64 (11)	C16—C15—C13	111.5 (5)
N3^i^—Hg—Cl2	115.22 (11)	C18—C15—C13	110.7 (5)
N1—Hg—Cl1	102.02 (10)	C16—C15—C17	107.9 (6)
N3^i^—Hg—Cl1	103.38 (10)	C18—C15—C17	107.8 (6)
Cl2—Hg—Cl1	126.35 (7)	C13—C15—C17	105.7 (6)
C1—N1—C2	105.5 (4)	C15—C16—H16A	109.5
C1—N1—Hg	125.2 (3)	C15—C16—H16B	109.5
C2—N1—Hg	129.3 (3)	H16A—C16—H16B	109.5
C1—N2—C7	106.8 (4)	C15—C16—H16C	109.5
C1—N2—C8	128.6 (4)	H16A—C16—H16C	109.5
C7—N2—C8	124.4 (4)	H16B—C16—H16C	109.5
N1—C1—N2	112.5 (4)	C15—C17—H17A	109.5
N1—C1—C9	123.9 (4)	C15—C17—H17B	109.5
N2—C1—C9	123.5 (4)	H17A—C17—H17B	109.5
N1—C2—C3	131.1 (5)	C15—C17—H17C	109.5
N1—C2—C7	108.9 (4)	H17A—C17—H17C	109.5
C3—C2—C7	120.0 (5)	H17B—C17—H17C	109.5
C4—C3—C2	117.6 (6)	C15—C18—H18A	109.5
C4—C3—H3A	121.2	C15—C18—H18B	109.5
C2—C3—H3A	121.2	H18A—C18—H18B	109.5
C3—C4—C5	120.8 (5)	C15—C18—H18C	109.5
C3—C4—H4A	119.6	H18A—C18—H18C	109.5
C5—C4—H4A	119.6	H18B—C18—H18C	109.5
C6—C5—C4	122.5 (5)	C19—N3—C20	106.0 (4)
C6—C5—H5A	118.7	C19—N3—Hg^ii^	123.8 (3)
C4—C5—H5A	118.7	C20—N3—Hg^ii^	129.2 (3)
C5—C6—C7	116.5 (6)	C19—N4—C25	106.3 (5)
C5—C6—H6A	121.7	C19—N4—C26	127.3 (5)
C7—C6—H6A	121.7	C25—N4—C26	126.4 (5)
N2—C7—C6	131.2 (5)	N3—C19—N4	112.5 (4)
N2—C7—C2	106.3 (4)	N3—C19—C11	125.0 (5)
C6—C7—C2	122.5 (5)	N4—C19—C11	122.4 (5)
N2—C8—H8A	109.5	N3—C20—C21	131.9 (3)
N2—C8—H8B	109.5	N3—C20—C25	108.0 (3)
H8A—C8—H8B	109.5	C21—C20—C25	120.0
N2—C8—H8C	109.5	C20—C21—C22	120.0
H8A—C8—H8C	109.5	C20—C21—H21B	120.0
H8B—C8—H8C	109.5	C22—C21—H21B	120.0
C14—C9—C10	119.3 (4)	C23—C22—C21	120.0
C14—C9—C1	119.0 (4)	C23—C22—H22B	120.0
C10—C9—C1	121.6 (4)	C21—C22—H22B	120.0
C11—C10—C9	119.5 (4)	C22—C23—C24	120.0
C11—C10—Br1	118.6 (3)	C22—C23—H23B	120.0
C9—C10—Br1	121.8 (4)	C24—C23—H23B	120.0
C12—C11—C10	120.4 (4)	C23—C24—C25	120.0
C12—C11—C19	118.0 (5)	C23—C24—H24B	120.0
C10—C11—C19	121.6 (5)	C25—C24—H24B	120.0
C11—C12—C13	121.3 (5)	N4—C25—C24	132.8 (3)
C11—C12—H12A	119.4	N4—C25—C20	107.2 (3)
C13—C12—H12A	119.4	C24—C25—C20	120.0
C12—C13—C14	117.7 (5)	N4—C26—H26D	109.5
C12—C13—C15	120.7 (5)	N4—C26—H26E	109.5
C14—C13—C15	121.5 (4)	H26D—C26—H26E	109.5
C9—C14—C13	121.6 (4)	N4—C26—H26F	109.5
C9—C14—H14A	119.2	H26D—C26—H26F	109.5
C13—C14—H14A	119.2	H26E—C26—H26F	109.5
			
C2—N1—C1—N2	−0.7 (5)	C11—C12—C13—C15	−174.2 (5)
Hg—N1—C1—N2	178.9 (3)	C10—C9—C14—C13	−2.4 (8)
C2—N1—C1—C9	−178.7 (4)	C1—C9—C14—C13	179.1 (5)
Hg—N1—C1—C9	0.8 (6)	C12—C13—C14—C9	−0.7 (8)
C7—N2—C1—N1	1.1 (5)	C15—C13—C14—C9	176.1 (5)
C8—N2—C1—N1	−173.1 (5)	C12—C13—C15—C16	−20.5 (8)
C7—N2—C1—C9	179.1 (4)	C14—C13—C15—C16	162.8 (5)
C8—N2—C1—C9	4.9 (8)	C12—C13—C15—C18	−147.0 (5)
C1—N1—C2—C3	178.3 (5)	C14—C13—C15—C18	36.3 (8)
Hg—N1—C2—C3	−1.2 (8)	C12—C13—C15—C17	96.5 (6)
C1—N1—C2—C7	0.0 (5)	C14—C13—C15—C17	−80.2 (7)
Hg—N1—C2—C7	−179.5 (3)	C20—N3—C19—N4	−0.1 (7)
N1—C2—C3—C4	−179.2 (5)	Hg^ii^—N3—C19—N4	169.2 (4)
C7—C2—C3—C4	−1.1 (7)	C20—N3—C19—C11	−176.2 (5)
C2—C3—C4—C5	−0.1 (8)	Hg^ii^—N3—C19—C11	−6.9 (8)
C3—C4—C5—C6	1.4 (8)	C25—N4—C19—N3	−1.0 (8)
C4—C5—C6—C7	−1.4 (8)	C26—N4—C19—N3	177.1 (8)
C1—N2—C7—C6	−179.8 (5)	C25—N4—C19—C11	175.2 (6)
C8—N2—C7—C6	−5.3 (8)	C26—N4—C19—C11	−6.6 (12)
C1—N2—C7—C2	−1.0 (5)	C12—C11—C19—N3	80.3 (8)
C8—N2—C7—C2	173.5 (4)	C10—C11—C19—N3	−99.4 (7)
C5—C6—C7—N2	178.9 (5)	C12—C11—C19—N4	−95.5 (7)
C5—C6—C7—C2	0.2 (7)	C10—C11—C19—N4	84.8 (8)
N1—C2—C7—N2	0.6 (5)	C19—N3—C20—C21	176.7 (4)
C3—C2—C7—N2	−177.9 (4)	Hg^ii^—N3—C20—C21	8.2 (7)
N1—C2—C7—C6	179.5 (4)	C19—N3—C20—C25	1.1 (5)
C3—C2—C7—C6	1.0 (7)	Hg^ii^—N3—C20—C25	−167.4 (3)
N1—C1—C9—C14	54.6 (7)	N3—C20—C21—C22	−175.2 (5)
N2—C1—C9—C14	−123.2 (5)	C25—C20—C21—C22	0.0
N1—C1—C9—C10	−123.9 (5)	C20—C21—C22—C23	0.0
N2—C1—C9—C10	58.3 (7)	C21—C22—C23—C24	0.0
C14—C9—C10—C11	3.6 (8)	C22—C23—C24—C25	0.0
C1—C9—C10—C11	−177.8 (5)	C19—N4—C25—C24	−175.9 (4)
C14—C9—C10—Br1	−173.0 (4)	C26—N4—C25—C24	5.9 (12)
C1—C9—C10—Br1	5.5 (7)	C19—N4—C25—C20	1.7 (7)
C9—C10—C11—C12	−1.8 (8)	C26—N4—C25—C20	−176.5 (8)
Br1—C10—C11—C12	175.0 (4)	C23—C24—C25—N4	177.4 (7)
C9—C10—C11—C19	177.9 (5)	C23—C24—C25—C20	0.0
Br1—C10—C11—C19	−5.4 (7)	N3—C20—C25—N4	−1.8 (5)
C10—C11—C12—C13	−1.4 (8)	C21—C20—C25—N4	−178.0 (5)
C19—C11—C12—C13	178.9 (5)	N3—C20—C25—C24	176.2 (4)
C11—C12—C13—C14	2.6 (8)	C21—C20—C25—C24	0.0

Symmetry codes: (i) −*x*+1, *y*+1/2, −*z*+1/2; (ii) −*x*+1, *y*−1/2, −*z*+1/2.

### Hydrogen-bond geometry (Å, º)

Cg1 is the centroid of the imidazole ring N1/N2/C1/C2/C7.

**Table:**

*D*—H···*A*	*D*—H	H···*A*	*D*···*A*	*D*—H···*A*
C3—H3*A*···N3^i^	0.95	2.65	3.459 (7)	144
C8—H8*A*···Cl2^ii^	0.98	2.71	3.643 (6)	160
C8—H8*B*···Cl1^iii^	0.98	2.82	3.719 (6)	152
C21—H21*B*···Cl1^ii^	0.95	2.77	3.616 (3)	149
C16—H16*B*···*Cg*1^ii^	0.98	2.91	3.671 (8)	135

Symmetry codes: (i) −*x*+1, *y*+1/2, −*z*+1/2; (ii) −*x*+1, *y*−1/2, −*z*+1/2; (iii) *x*+1, *y*, *z*.
